# New Approach Based on Compressive Sampling for Sample Rate Enhancement in DASs for Low-Cost Sensing Nodes

**DOI:** 10.3390/s141018915

**Published:** 2014-10-13

**Authors:** Francesco Bonavolontà, Massimo D'Apuzzo, Annalisa Liccardo, Michele Vadursi

**Affiliations:** 1 Dipartimento di Ingegneria Elettrica e delle Tecnologie dell'Informazione, Università degli Studi di Napoli Federico II, Via Claudio 21, Naples 80125, Italy; E-Mails: francesco.bonavolonta@unina.it (F.B.); dapuzzo@unina.it (M.D.A.); 2 Dipartimento di Ingegneria, Università di Napoli “Parthenope”, Centro Direzionale di Napoli, Isola C4, Naples 80143, Italy; E-Mail: michele.vadursi@uniparthenope.it

**Keywords:** compressive sampling, high time resolution ADC, sample rate, random sampling

## Abstract

The paper deals with the problem of improving the maximum sample rate of analog-to-digital converters (ADCs) included in low cost wireless sensing nodes. To this aim, the authors propose an efficient acquisition strategy based on the combined use of high-resolution time-basis and compressive sampling. In particular, the high-resolution time-basis is adopted to provide a proper sequence of random sampling instants, and a suitable software procedure, based on compressive sampling approach, is exploited to reconstruct the signal of interest from the acquired samples. Thanks to the proposed strategy, the effective sample rate of the reconstructed signal can be as high as the frequency of the considered time-basis, thus significantly improving the inherent ADC sample rate. Several tests are carried out in simulated and real conditions to assess the performance of the proposed acquisition strategy in terms of reconstruction error. In particular, the results obtained in experimental tests with ADC included in actual 8- and 32-bits microcontrollers highlight the possibility of achieving effective sample rate up to 50 times higher than that of the original ADC sample rate.

## 1. Introduction

In recent years, embedded systems (such as microcontrollers, field programmable gated arrays, digital signal processors and so on) have been playing a fundamental role in metrological applications. The availability of integrated systems capable of digitizing, processing and transmitting measurement results offers the opportunity of realizing nodes for distributed and/or portable measurement systems characterized by reduced costs and good performance. Typical application examples are smart meters for energy billing or analysis of electrical power quality [1–4], monitoring of environmental quantities of interest [5–8], control of complex production process [9–11].

Architectures based on successive approximation registers are usually chosen for the Data Acquisition Section (DAS), mainly based on the Analog to Digital Converter (ADC) embedded in the measurements nodes, due to their straightforward implementation and a nominal vertical resolution that is suitable for most of the considered application. Nevertheless, some specific solutions for band-pass signals [12–14] and some dedicated solutions exploiting more performing ADCs (ΣΔ or flash converters) are also available on the market [15].

The most significant parameters commonly used for characterizing and determining the performance of the DAS are:

- Nominal vertical resolution: usually expressed in bits, it defines how many distinct output codes the DAS can produce; depending on the specific architecture of the ADC, typical resolution varies between 10 and 14 bits;
- Maximum sample rate: it defines the capability of the DAS of rapidly sampling and converting the input signal and directly determines the maximum spectral component that can be alias‐free sampled. Typical actual values for low cost microcontrollers range from few tens of kilohertz up to five MHz;
- Memory depth: combined with the sample rate, it determines the maximum observation interval the microcontroller can acquire for successive processing. Values from few kilobytes up to some megabytes are usually found;
- Input bandwidth: determines the maximum frequency of spectral components that the ADC can receive as input without significant distortion; to assure alias-free digitization of the input signal, it is usually set no higher than half maximum sample rate, even though some solutions dedicated to digital down conversion provide larger bandwidth [16].

For distributed measurement systems consisting of distributed acquisition nodes and a central computing unit that processes measurement data, improving the performance of the embedded DAS, in terms of sample rate enhancement, can be crucial for the improvement of the whole measurement system. To this aim, the paper presents a novel acquisition strategy, based on compressive sampling (CS), which permits to increase the maximum sample rate of DAS integrated in low‐cost microcontrollers.

CS is a recent and attractive sampling approach capable of assuring reliable reconstruction of signals of interest from a very reduced number of acquired samples, provided that some conditions about the signal and sampling scheme are met [17]. Some papers recently focused their attention on the possibility of exploiting CS to enhance the performance of ADC in terms of sample rate. In particular, in [18] CS is used to extend the traditional equivalent time sampling (ETS) scheme in order to reconstruct the input signal with a higher time resolution. However, a number of periods of the input signal much higher than those successively reconstructed are involved, which poses severe constraints on the stability of the time-base. Such problem has been solved for random sampling CS-based ADC in [19] through an *ad*-*hoc* new circuit. An alternative is represented by CS-based ADCs exploiting random demodulation [20], which have been shown to have good performance for most measurement applications [21].In particular, a significant improvement in terms of sample rate has been obtained, though at the expense of architectural complexity, due to the presence of the analog mixing of the input signal with a pseudo-random sequence.

Differently from the above-mentioned solutions, the method proposed hereinafter does not require any hardware modification (such as external clock circuits and/or analog mixing stages) to increase the sample rate and turns out to be the optimal solution for the majority of already available ADCs integrated in embedded systems. The proposed acquisition strategy permits, in fact, to achieve a higher time resolution when digitizing a signal included in the ADC bandwidth in real-time, by combining the already available hardware section (constituted by the traditional ADC and the high-resolution time-basis) with a proper software procedure, which provides a suitable random sequence of sampling instants and reconstructs the signal of interest according to the CS theory.

## 2. Problem Statement

Taking advantage of some attractive features of the CS theory, a new method is proposed in the following section with the aim of improving the nominal sample rate of ADCs. Although the proposed method is general, it proves particularly advantageous when applied to ADC modules included in low cost microcontrollers (MCs). The availability of a high-resolution time‐basis (such as that generated from the fundamental clock frequency for MCs) allows, in fact, finely defining a suitable random sequence of the sampling instants capable of assuring the reliable successive signal reconstruction. For the sake of clarity, the key idea underlying the method is described and compared both to the traditional and compressive sampling approach.

### 2.1. Traditional Sampling Approach

**Figure 1.**
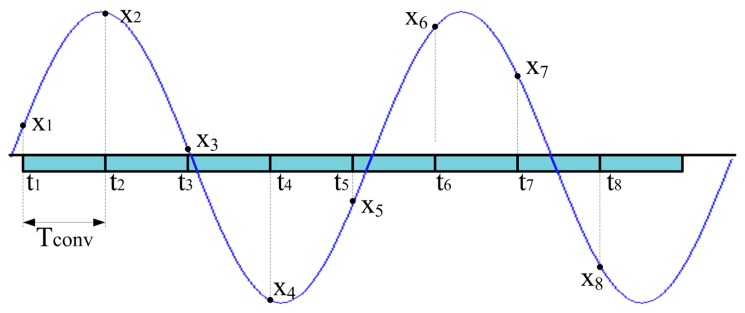
Traditional approach for signal sampling; samples of the input signal of interest are uniformly digitized with constant period equal to *T_conv_*.

In Figure 1 the traditional sampling approach, adopted by the majority of ADCs, is shown. The ADC operates at its highest sampling rate and input signal samples are uniformly taken with a sampling period equal to *T_conv_*, which is the time interval required by the ADC to digitize (*i.e.*,to sample and convert) a single sample. Alias-free sampling can be assured on a signal whose maximum spectral content is lower than 1/(2*T_conv_*). It is useful to highlight that in the case of low-cost microcontrollers, the limited memory depth (usually shorter than 10,000 samples) permits to save only short-time records of the input signal.

### 2.2. CS-Based Sampling Approach

**Figure 2.**
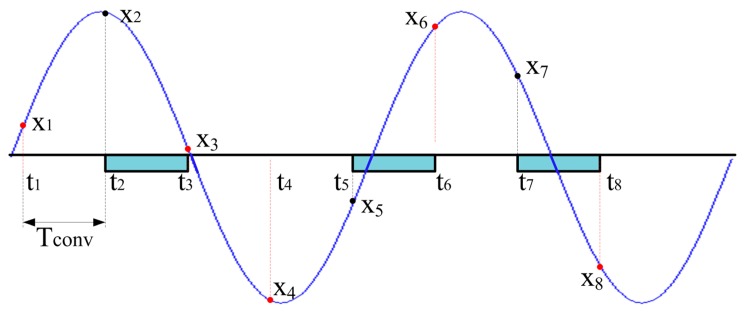
Sampling strategy based on CS; only few samples are randomly digitized from which the input signal can be reconstructed with constant sampling period equal to *T_conv_*.

The authors recently faced the considered problem thanks to a suitable sampling approach based on CS [22–24]. As shown in Figure 2, if the samples (indicated by black dots) are randomly acquired throughout the observation interval, the signal of interest can accurately be reconstructed (samples marked by red dots) as it had been continuously digitized with a sampling period equal to *T_conv_*.

It is worth noting that the desired reconstruction is achieved, starting from a very limited number of random signal samples. It was shown that accurate reconstruction can be gained, with a compression ratio up to 98% for multicomponent signals (*i.e.*, 10,000 samples input signals were recovered starting from 200 acquired random samples).

### 2.3. Proposed Sample Rate Improvement

**Figure 3.**
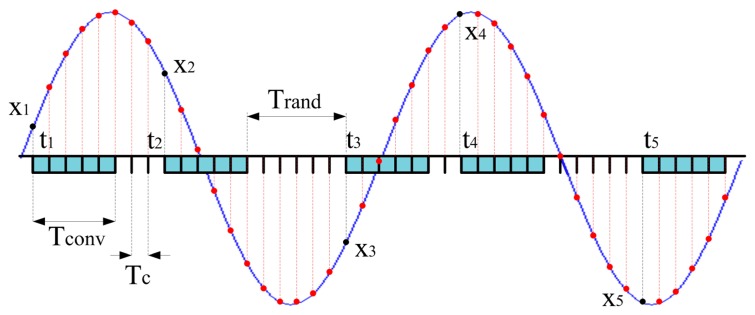
Proposed acquisition strategy based on CS; thanks to the availability of a suitable time-basis, the input signal can be reconstructed with constant sampling period equal to *T_c_* < *T_conv_*.

A new method (in the following referred to as new acquisition strategy) based on CS has been defined and implemented for increasing the effective sample rate of embedded DAS. The availability of a suitable time-basis allows finely setting the random sampling instants (*i.e.*, the time instants the ADC starts to convert a single sample, which are marked as black dots in Figure 3), with a time resolution equal to *T_c_*. Even though the conversion of a single sample takes a time *T_conv_* greater than *T_c_* (*T_conv_* = 5*T_c_* in the example shown in Figure 3), the proposed approach assures that the input signal will finally be reconstructed (red dots) at an effective sample rate equal to 1/*T_c_*. As it can be expected, the only constraint is that the time difference between two successive actual sampling instants should be greater than *T_conv_*.

## 3. Proposed Sampling Approach

**Figure 4.**
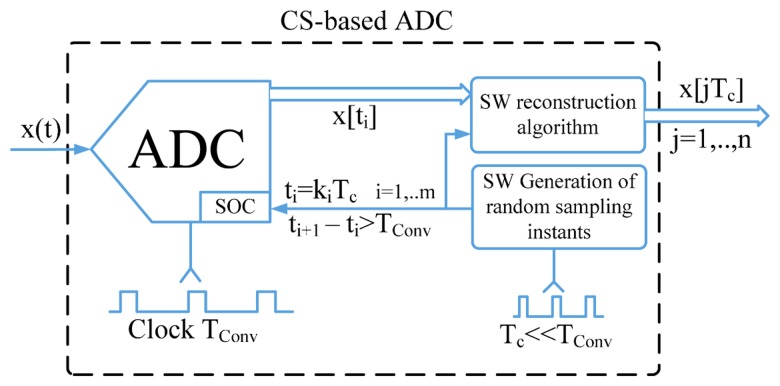
Block diagram of proposed solution for improving ADC sample rate.

To improve the sample rate of ADC in low‐cost embedded systems, the traditional hardware for analog-to-digital conversion has been complemented with a proper digital signal processing mandated to generate the random sequence of sampling instants and reconstruct the signal of interest from the acquired samples (Figure 4). In particular, the sequence of the random sampling instants is determined as a multiple of a high-resolution time-basis, *T_c_*, and exploited to control the start of conversion (SOC) signal of the ADC. The sequence of considered instants and the corresponding samples (digitized at lower rate, equal to 1/*T_conv_*, by the ADC) are given as input to the CS-based algorithm for the successive signal reconstruction, with a time resolution equal to that of the adopted time-basis. Specific details about the determination of the random sampling instants along with some guidelines for the reconstruction algorithm are given in the following.

### 3.1. Sampling Instants Determination

The first step of the new acquisition strategy is the determination of the actual sampling instants. According to the random sampling approach [25], the sampling instants *t_i_* are randomly chosen throughout the considered observation interval *T_w_* equal to *n* times *T_c_*. The key idea underlying the proposed sampling strategy is that the considered instants can be expressed as an integer multiple of the high-resolution time-basis *T_c_*:

(1) $$t_{i}=k_{i}T_{c},k_{i}\in [0,n-1]\;\text{and}\;i=1,..,m$$

This way, the final signal reconstruction will be obtained with the same time resolution, thus granting a suitable enhancement of the nominal ADC sample rate. In order to assure proper operations of the CS-based ADC, the sampling instants *t_i_* have to satisfy the following expression (Figure 3)

(2) $$t_{i}=t_{i-1}+T_{\text{conv}}+T_{\text{rand}}$$

where *T_rand_* is the random interval between the end of a conversion and the start of the successive one; the considered constraint assures that no new conversion will start until the pending one is over. Moreover, a specific software procedure has been implemented in order to assure the generation of a pseudo‐random sequence of multiples *k_i_* capable of assuring the full coverage of the observation with a suitable grade of randomness. In particular, let *fr* be the value of the ratio between *T_conv_* and *T_c_*; the pseudo‐random sequence generator has to assure the determination of *m* sampling instants within the interval from 0 up to *nT_c_*, each of which far at least *frT_c_* from the successive one. To this aim, the procedure enlists the following steps:

1. Evaluation of a suitable acceptance threshold *tsh*, equal to *m*/*n* (only *m* sampling instants among *n* possible values have to be determined) in the first iteration;
2. Current sampling instant index *k_i_* is initially equal to 0;
3. A pseudo‐random number is generated according to uniform random distribution within the interval from 0 up to 1;
4. If the obtained pseudo‐random number is lower than the acceptance threshold, then *k_i_* is retained in sampling sequence and its value is updated by adding *fr*;
5. If the obtained pseudo‐random number is greater than the acceptance threshold, then *k_i_* is dropped and its value is incremented by one;
6. If the number of sampling instants included in the sequence is lower than *m* and *k_i_* is lower than *n−1* return to step 3;
7. If *k_i_* is not lower than *n* but the sampling instants sequence is not yet full, a new acceptance threshold has to be calculated; this is particularly likely when the value *mfr* is close to *n*. To assure a fast convergence of the procedure, the authors adopted a threshold increment of 20%, *i.e.*, the new value of *tsh* is 1.2 times the old *tsh* value; once updated the *tsh* value, return to step 2;
8. If the number of sampling instants included in the sequence is equal to *m* and *k_i_* value is lower than *n*−1, the sampling instants sequence is complete and can be adopted to acquire the samples of the input signal.

With regard to *T_conv_*, since it is generated from the same fundamental clock, it usually is a multiple of the adopted time-basis; if this is not the case, the first multiple of *T_c_* immediately greater than *T_conv_* is used, thus granting that condition (2) always holds.

According to the CS approach [26], the relation between the sequence of acquired samples ***y*** ∈ ℝ^m^ and the input signal of interest ***x*** ∈ ℝ^n^ can be expressed as

(3) y=Φx

where **Φ** ∈ ℝ**^mxn^** is the so-called sampling matrix. The values *k_i_* turn out to be the indexes of the columns of the random sampling matrix **Φ** containing non-null entries, whose value is equal to one. For the sake of the clarity, let us suppose to be interested in recovering an 8‐samples input signal ***x*** from 3 samples ***y*** acquired in time domain. Assuming that *T_conv_* lasts 2*T_c_*, the equations system (3) can be rewritten as:

(4) $$\left[\begin{array}{c} y(0)\\ y(1)\\ y(2) \end{array}\right]=\left[\begin{array}{cccccccc} 1 & 0 & 0 & 0 & 0 & 0 & 0 & 0\\ 0 & 0 & 0 & 0 & 1 & 0 & 0 & 0\\ 0 & 0 & 0 & 0 & 0 & 0 & 0 & 1 \end{array}\right]*\left[\begin{array}{c} x(0)\\ x(1)\\ \ldots \\ x(6)\\ x(7) \end{array}\right]=\left[\begin{array}{c} x(0)\\ x(4)\\ x(7) \end{array}\right]$$

### 3.2. Sensing Matrix Determination

Being usually *m* ≪ *n* (*i.e.*, the number of equations lower than that of the unknowns), the problem of recovering ***x*** from the acquired samples through the equations system (3) results ill-posed [27] and cannot be solved via traditional approaches based on least squares minimization. The problem can, fortunately, be bypassed if a similar system can be written in the form

(5) y=Af

where **A** ∈ ℝ**^mxn^** is the so-called sensing matrix and ***f*** ∈ ℝ**^n^** is a sparse vector. Avector is said to be *S*-sparse if only *S* of its components are (significantly) greater than zero. Expressing the signal of interest in terms of its sparse representation turns out to be mandatory; to this aim, a suitable orthonormal basis **Ψ** has to be found, according to

(6) x=Ψf

With reference to the measurement applications considered in Section I, most of the desired signals are characterized by sparse representations in the frequency domain. This way, the Fourier basis has been chosen and the corresponding matrix **Ψ**, whose entries are defined as

(7) $$\psi_{i,p}=\frac{1}{\sqrt{n}}e^{j\frac{2\pi }{n}i\cdot p}\forall i,p\in [0,..,n-1]$$

has been adopted as transformation matrix. By comparing Equations (3) and (6), the sensing matrix can be expressed,

(8) A=ΦΨ

Thanks to the specific choice of the sampling matrix, the matrix **A** can be generated as a submatrix of **ψ**, thus reducing the computational burden of the method, since **A** doesn't have to be calculated from actual multiplications. It is, in fact, evaluated as the rows of the matrix **ψ**, whose indexes match those the sampling instants *k_i_*, thus granting its possible implementation also on devices characterized by reduced memory depth. With reference to the sampling matrix in Equation (4), the corresponding sensing matrix is given by:

(9) $$\text{A}=\left[\begin{array}{cccc} \frac{1}{\sqrt{8}} & \frac{1}{\sqrt{8}} & \ldots & \frac{1}{\sqrt{8}}\\ \frac{1}{\sqrt{8}} & \frac{1}{\sqrt{8}}e^{j\frac{2\pi }{8}4} & \ldots & \frac{1}{\sqrt{8}}e^{j\frac{2\pi }{8}28}\\ \frac{1}{\sqrt{8}} & \frac{1}{\sqrt{n}}e^{j\frac{2\pi }{8}7} & \ldots & \frac{1}{\sqrt{8}}e^{j\frac{2\pi }{8}49} \end{array}\right]$$

### 3.3. Sparse Solution Evaluation

Even though the equations system in Equation (5) is still underdetermined, the sparsity of ***f*** can be exploited to find a suitable solution [28,29]. More specifically, it is possible to recover ***x*** by solving the following optimization problem

(10) $$\begin{array}{cc} \hat{\text{f}}=\underset{\text{f}}{\arg\min}{\left\|\text{f}\right\|}_{1} & s.t.f\in \text{B}(y) \end{array}$$

where ‖·‖_1_ stands for the *l*_1_-norm (*i.e.*, the sum of the absolute values of the ***f*** components) and 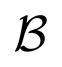
(***y***) is a proper constraint that assures the consistence with the samples ***y***. In particular, in the presence of noise-free samples, the feasible set 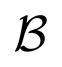
(***y***) can be expressed as

(11) B(y)={f:Af=y}

If the acquired samples have been contaminated with small amount of noise **ε** (such as the quantization noise) a better expression would be

(12) $$\text{B}(\text{y})=\{\text{f}:{\left\|Af-\text{y}\right\|}_{2}\leq \varepsilon \}$$

In other words, the best estimate ***f̂*** of the input signal spectrum turns out to be the sparse representation characterized by the minimum *l_1_*-norm. The use of *l_1_*-norm grants, in fact, that obtained solution will be sparse, a condition that is usually not met when least square minimization approaches are adopted. Moreover, the constraints (11) and (12) define the so-called feasible set and assure that the required estimate ***f̂*** is a solution (either absolute or approximated) of the Equation (5).

### 3.4. Input Signal Recovering

Once the solution ***f̂*** is obtained, the input signal of interest can easily be recovered by means of Equation (6)

(13) $$\hat{x}=\Psi \hat{f}$$

It is worth reminding that the time support of the recovered signal is the whole observation interval *T_w_* and its quantization is related to the resolution of the time-basis adopted to define the sampling instants.

Finally, some considerations have to be drawn about the number of random digitized samples. In particular, it has been demonstrated [30] that the number of samples *m* has to meet the following condition:

(14) $$m\geq S\mu {\left(\Phi ,\Psi \right)}^{2}\log n$$

where *μ*(**Φ**, **Ψ**) is the so-called coherence between the matrices **Φ** and **Ψ**, defined as the quantity:

(15) $$\mu (\Phi ,\Psi )={\sqrt{n}}_{\overset{\max}{1\leq k,j\leq n}}\left|<\varphi_{k},\psi_{j}>\right|$$

and φ_k_ and ψ_j_ stand, respectively, for the *k*-th row and the *j*-th column of the matrices **Φ** and **Ψ** and <·.·> indicates the traditional inner product.

The coherence proves to be a fundamental parameter for the compression in determining the number of needed samples. The lower its value, the fewer the samples required for a reliable reconstruction of *f* and consequently of the original signal *x*. According to the choices made about **Φ** and **Ψ**, the coherence reaches the minimum allowed value, equal to 1, in the considered acquisition strategy, thus granting a proper reconstruction of an *S*‐components signal with about *S* log *n* random samples. On the contrary, as it can be expected, the higher the number *m* of acquired samples, the lower the reconstruction error. However, as described in the following section, once the sparsity of the input signal is known, acquiring a larger number of samples proves to be not advantageous, since no more improvement in the reconstruction is experienced, while increasing the computational burden worthlessly.

## 4. Numerical Results

To preliminarily assess the performance of the proposed sampling strategy, several tests have been executed by means of numerical simulations. The effect of the most influencing parameters, such as number of acquired samples *m*, ADC sample rate *f_conv_*, ADC vertical resolution *n_bit_*, signal-to-noise ratio *SNR*, jitter and input signal sparsity *S*, has been evaluated. Parameters value has been chosen as close as possible to those provided by the cheapest microcontrollers [31] or granted by most of the embedded systems [32] that are currently available on the market. Similar values will be selected in the successive actual experimental tests. With regard to the number of acquired samples *m*, it has always been lower than typically available memory depth. On the contrary, the number of samples *n* granted for the signal reconstruction has been even much greater than memory depth, thanks to the CS-based approach.

As an example, some of the obtained results are presented in the following. Unless otherwise indicated, the input signal for tests has been a pure unipolar sinusoidal signal whose full scale amplitude and frequency were equal respectively to 2*^nbit^*−1 codes and 5 kHz; 80 random samples have been digitized with an effective vertical resolution of 12 bits at an ADC sample rate *f_conv_* equal to 10 kS/s, and the signal has been reconstructed over a time sequence of 10,000 samples at an effective sample rate *f_c_* of 1MS/s. For the sake of clarity, the parameters values are summarized in Table 1.

The reconstruction error has been used to assess the performance of the acquisition strategy. According to what stated in [13], it is defined as:

(16) $$\varepsilon =\frac{\left\|\hat{x}-x\right\|}{\left\|x\right\|}\cdot 100$$

where *x̂* is the reconstructed signal and *x* is the original one.

### 4.1. ADC Sample Rate and Vertical Resolution

A first set of tests aimed at verifying the dependence of the performance of the proposed acquisition strategy on the ADC conversion period and effective number of bits. Several nominal values of ADC sample rate *f_conv_* and vertical resolution *n_bit_* have been taken into account.

**Figure 5.**
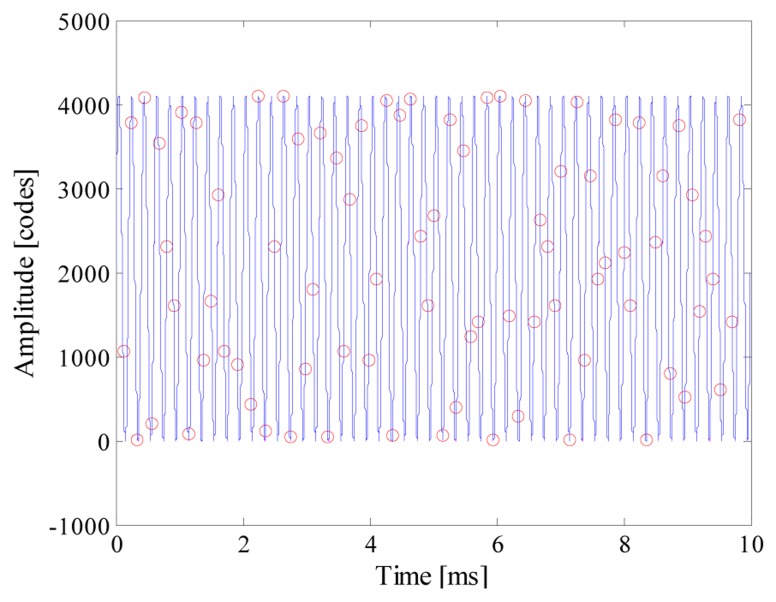
Example of input signal, acquired samples (red circles), and reconstructed signal.

**Figure 6.**
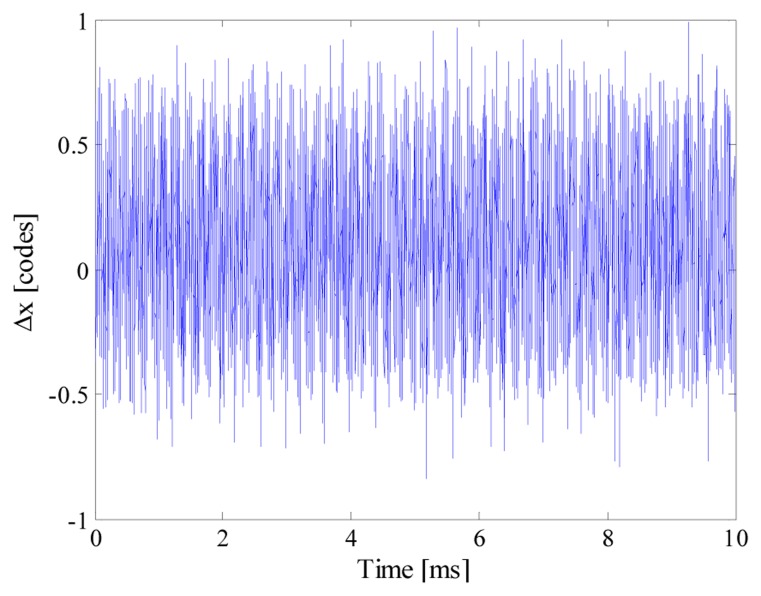
Point-by-point differences between reconstructed and input signal.

As an example, Figure 5 shows the input signal, the acquired samples and the reconstructed signal (which completely overlies the input signal) when *n_bit_* and *f_conv_* were equal respectively to 12 and 10 kS/s. It is worth noting that 80 samples are randomly taken throughout 50 periods of the input signal; the acquired sequence clearly violates the Nyquist theorem. To better appreciate the performance of the proposed acquisition strategy, point‐by‐point differences Δ*x* between the reconstructed signal *x̂* and the input signal *x* is shown in Figure 6. Differences greater than 1 code have never been found, thus assuring a reconstruction error as low as 0.007%.

**Figure 7.**
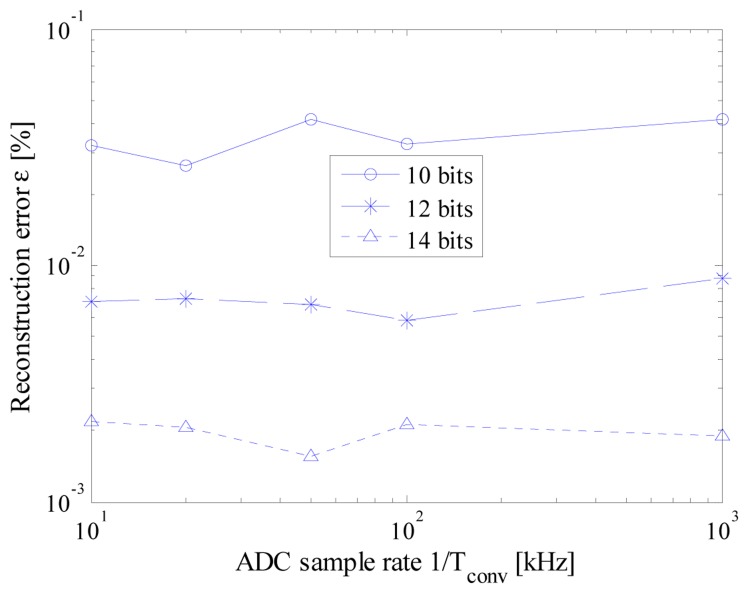
Reconstruction error *versus* the ADC sample rate for different values of ADC vertical resolutions; markers associated with an ADC nominal sample rate of 1MS/s accounts for traditional CS approach.

Some results of the executed tests are summarized in Figure 7. As it can be seen, the performance of the acquisition strategy turned out to be almost independent from the nominal ADC sample rate. This way, the desired acquisition can be carried out by exploiting the ADC with the lowest available sample rate. It is so possible to make the ADC working in less critical conditions, thus allowing to take advantage of most of its effective number of bits.

Moreover, for each test configuration in terms of *n_bit_* and *f_conv_*, reconstruction error proved to be lower than the associated least significant bit (LSB), thus assuring that no harmful artifacts have been introduced by the proposed strategy. Finally, to compare the performance of the proposed acquisition strategy with that granted by the traditional CS approach [20], the same test has been executed with a *t_conv_* equal to *t_c_* for each value of *n_bit_*. The obtained values (*i.e.*, the markers corresponding to a nominal ADC sample rate of 1 MHz in Figure 7) highlighted that no significant difference can be appreciated whatever the vertical resolution of ADC; this behavior can be easily explained if the equations system (5) is taken into account. Even though the authors have defined a suitable procedure for the generation of the random sequence of sampling instants, this choice involves no significant differences in solving the system (5). In other words, any random sequence is as good as any other from a theoretical point of view; only a negligible degradation in the mean performance is experienced, due to the time difference constraint (2) that slightly reduce the possible randomness of the sequence indexes. Similar considerations hold also in the other investigated test conditions.

### 4.2. Noise

**Figure 8.**
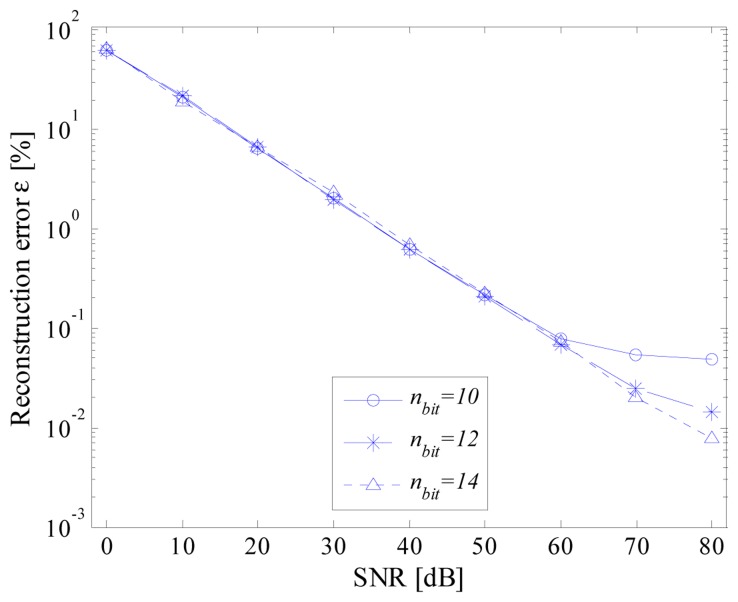
Reconstruction error *versus* SNR; for different values of effective number of bits, *n_bit_*.

The influence of the noise on the performance has successively been investigated. For each value of SNR, 1000 pseudo‐random sequences generated according to an additive white Gaussian noise (AWGN) have been added to the input signal; the average value of reconstruction errors have been evaluated. As an example, some results, obtained for different values of effective number of bits, are shown in Figure 8. As expected, the higher the SNR, the better the performance of the proposed acquisition strategy. In particular, reconstruction errors similar to those given in Figure 7 have been achieved only for the higher values of SNR.

### 4.3. Number of Acquired Samples and Effective Sample Rate

**Figure 9.**
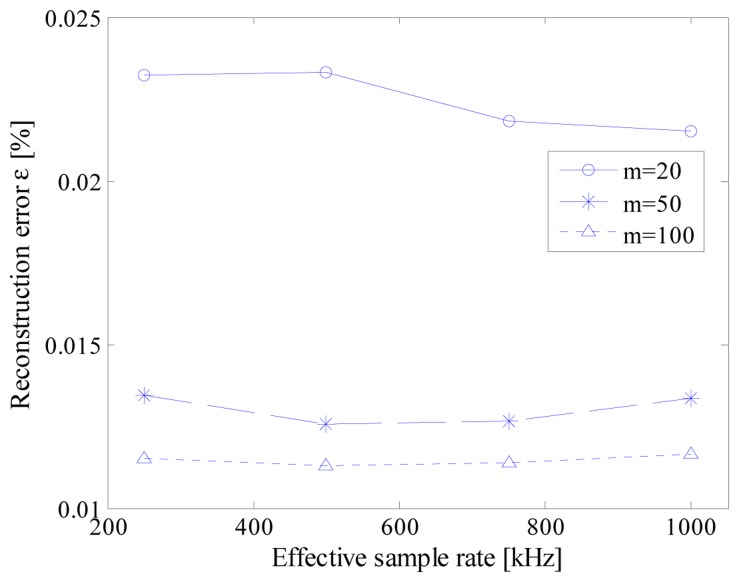
Reconstruction error *versus* the effective sample rate *f_c_* for different values of number *m* of randomly acquired samples.

A number tests have then been performed for different combinations both of number of random samples *m* and effective sampling frequency *f_c_*. As an example, some results are summarized in Figure 9. As it can be appreciated, the difference between reconstructed and original signal is always lower than 1LSB, including when only 20 random samples are acquired, *i.e.*, in the presence of a compression ratio $\left(1-\frac{m}{n}\right)\cdot 100$ equal to 99.8%.

### 4.4. Jitter

**Figure 10.**
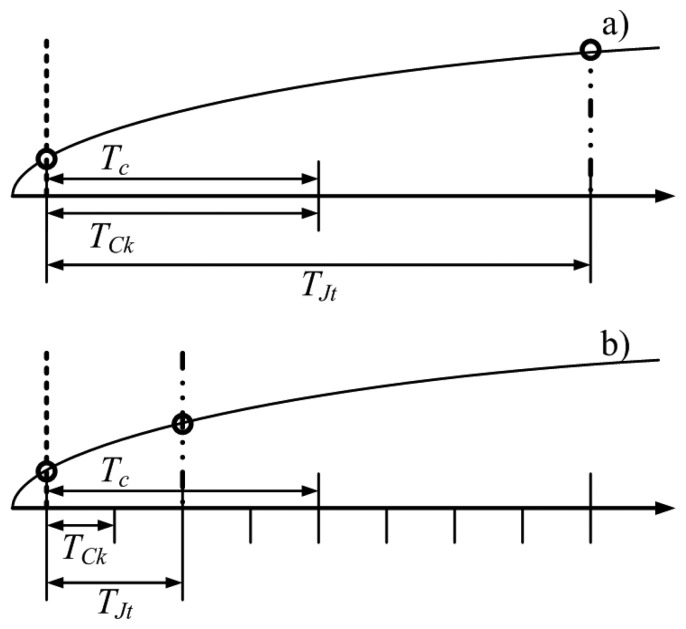
Effect of instruction cycle jitter for different values of *f_Ck_*/*f_c_*.

As stated above, all the clock signals (included the high-resolution time-basis adopted by the proposed acquisition strategy) of an embedded system are derived from a fundamental clock, usually referred to as instruction cycle clock, *f_Ck_*. Due to the specific architecture of the microcontroller and the software implementation of the time-basis, a random difference between the nominal sampling instant and the effective one can occur. Such difference can be expressed in terms of number instruction cycles and typically assume integer values within 0 and 10 [32]. In other words, from the nominal SOC instant to its actual execution, a random number of instruction cycles could occur, due to latency or uninterruptable instructions problems. It is worth noting that this drawback can be mitigated, but not completely eliminated, and acts as a jitter on the high-resolution time-basis. Moreover, the actual jitter of the fundamental clock can be neglected in the following analysis, since its value is much lower than that associated with the instruction cycles. For the sake of the clarity, Figure 10a,b shows the actual SOC events (point-dashed lines) associated with a difference of two instruction cycles (*T_Jt_*) from the nominal SOC event (dashed line) in the presence of ratios *f_Ck_*/*f_c_* equal respectively to 1 and 4; the effect of the jitter on the actual digitized sample (circle marker) on the input signal (full line curve) is clearly reduced.

**Figure 11.**
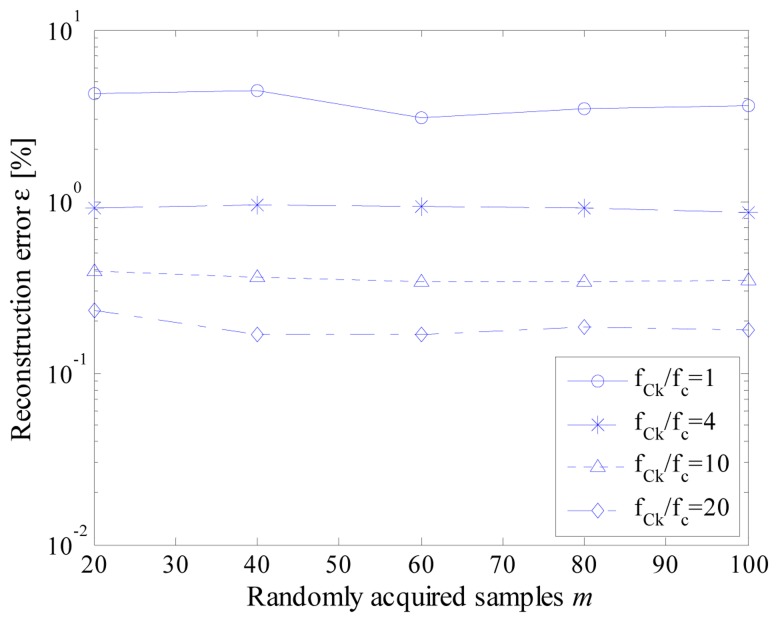
Reconstruction error *versus* the number *m* of randomly acquired samples for different values of the ratio *f_Ck_*/*f_c_*. when jitter equal to 10.

**Figure 12.**
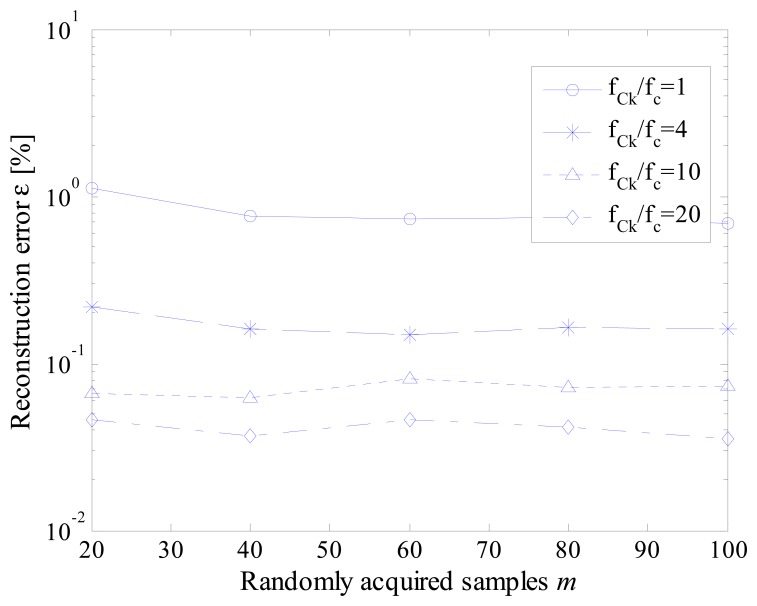
Reconstruction error *versus* the number *m* of randomly acquired samples for different values of the ratio *f_Ck_*/*f_c_*. when jitter equal to 2.

To analyze the effect of jitter, several scenarios have been simulated in terms of different values of ratio between instruction cycle clock frequency and effective sample rate. As an example, obtained results are presented in Figures 11 and 12 for jitter values of 10 (worst case) and 2 (reduced jitter) instruction cycles, respectively.

As it can be appreciated in Figure 11, 10 instruction cycles jitter highly degrades the performance of the proposed acquisition strategy. As expected, the worst results have been experienced when the effective sample rate matched the instruction cycle clock frequency; in this case, a difference with respect to the nominal value up to ten sampling instants can occur. Better results have been obtained for higher values of the ratio *f_Ck_*/*f_c_*. However, values of reconstruction error never lower than 0.1% has been encountered. The performance of the proposed acquisition strategy improves if the jitter is reduced down to 2 instruction cycles (Figure 12). Reconstruction errors of few hundredths can, in fact, be assured with a suitable level of ratio *f_Ck_*/*f_c_*.

### 4.5. Input Signal Sparsity

Finally, the reconstruction error has been evaluated *versus* different values of number of acquired samples and number of spectral components included in the input signal (*i.e.*, the signal sparsity in frequency domain). Specific test parameters are presented in Table 2. As for the sparsity, its maximum value has been chosen according to Equation (14), once defined the highest number of random acquired samples. For each value of the signal sparsity 1000 numerical input signals have been generated by adding *S* spectral components whose amplitude, phase and location within the Nyquist band have been randomly selected. For each test configuration, minimum, average and maximum values of the reconstruction errors have been calculated in terms of *m* and *S*; some of the results are reported in Table 3.

As it can be expected, the higher the spectral content of the input signal, the higher the number of samples that have to be acquired in order to accurately reconstruct the signal. However, values of reconstruction error up to 10% has been experienced also when the input signal has been recovered from 100 random samples; this is mainly due to the effect of the considered jitter on spectral components characterized by higher frequency. This way, either the use of instruction cycle clocks with higher values of frequency or a greater number of random acquired samples is advisable to further mitigate this harmful effect.

## 5. Experimental Tests

**Figure 13.**
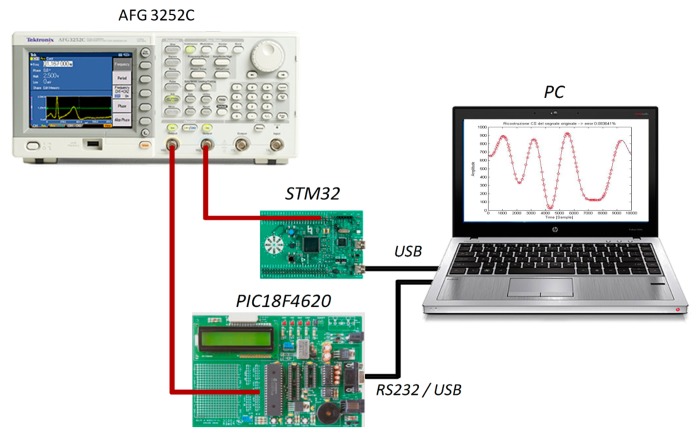
Block diagram of the adopted measurment station.

A number of tests have finally been executed to assess the performance of the proposed acquisition strategy on two different cost-effective hardware architectures, characterized, respectively, by 8- and 32-bits core microcontrollers and specifications very close to those presented in Sections 1 and 4. A suitable measurement station has been setup (Figure 13), which includes:

- A microcontroller acting as DAS (either 8- or 32-bits);
- A dual‐channel arbitrary function generator AFG3252C (maximum output frequency 240 MHz, 14 bits vertical resolution, 128 kSamples memory depth) by Tektronix;
- A personal computer mandated to (i) generate the random sequence of sampling instants; (ii) transmit it to the low-cost DAS; (iii) receive back the acquired samples and (iv) process them by means of a free tool (namely CVX [33] and working in MATLAB™ environment).

Input signals characterized by several sparsity values have been taken into account. With specific regard to signals different from pure sinusoidal tones, the so-called optimized multisine [34] has been adopted as test signal. The optimized multisine can be expressed as the sum of cosine waveform according to:

(17) $$x(t)=\sum_{h=1}^{S}A_{h}\cos(2\pi f_{h}t+\varphi_{h})$$

where *A_h_*, *f_h_*, and *φ_h_* stand for the amplitude, frequency and phase of the *h*-th spectral component, respectively. Their values can easily be combined to generate a multitone signal whose amplitude is tailored to the ADC full-scale range (3V and 5 V for 8- and 32-bits architecture respectively). In particular, for the considered application, the amplitude of the spectral components have been set to the same value in order to obtain a flat amplitude spectrum in the frequency region of interest. The phase of each component has been selected according to the criterion of crest factor (CF) minimization, thus assuring signals with suitable SNR in the whole observation interval. More specifically, Schroeder multisine [35] has been adopted; CF minimization was achieved by setting phase values according to the following expression:

(18) $$\varphi_{h}=-\frac{h(h-1)}{S}\pi$$

As for the tests conducted in simulations, the reconstruction error has been adopted as the performance indicator. The best estimate of the input signal *x* has been gained through either the traditional four parameters sine-fit [36] or multisine interpolation [37] of the reconstructed signal, according to the corresponding test.

### 5.1. Tests Conducted on 32-Bits Microcontroller

The performance of the acquisition strategy has first been assessed on a STM32F303VCTM by STMicroelectronics, a microcontroller based on ARM Cortex M4 core. It is characterized by a maximum instruction cycle frequency *f_Ck_* equal to 72 MHz, data memory depth of 40 KB, four ADCs with selectable vertical resolution (6, 8, 10, and 12 bit) and full scale of 3 V [32]. The available values of *T_conv_* consisted of the sum of:

- A constant term *T_SAR_* equal to (*n_Bit_* + 0.5) *T_Ck_* required for the execution of the operations of internal SAR ADC;
- A selectable term *T_Samp_* ranging from 1.5 up to 601.5 *T_Ck_* accounting for the sampling time [32].

Unless otherwise indicated, the input signal for tests has been a pure unipolar sinusoidal signal whose full scale amplitude and frequency were equal respectively to 3 Vpp and 1.2kHz; 100 random samples have been digitized with a nominal vertical resolution of 12-bits and the input signal has been reconstructed over a time sequence of 10,000 samples. The microcontroller was operated at its maximum instruction cycle frequency.

A first set of tests has been conducted to assess the influence of the nominal sample rate, *f_conv_*, on the reconstruction performance of the proposed strategy. As expected, the higher the value of *T_conv_*, (due to greater values of *T_Samp_*), the better the strategy performance, to the detriment of the ADC nominal sample rate. As an example, Table 4 summarizes the results obtained on a sinusoidal signal with frequency equal to 6 kHz, for effective sample rate ranging from 1MS/s and 12 MS/s: severe performance degradation has been experienced with the lowest value of *T_conv_* (195 ns). This is mainly due to limited duration of the associated sampling time; this way, largest *T_conv_* (usually 32 *T_Ck_*) have been adopted in the successive experimental tests.

**Figure 14.**
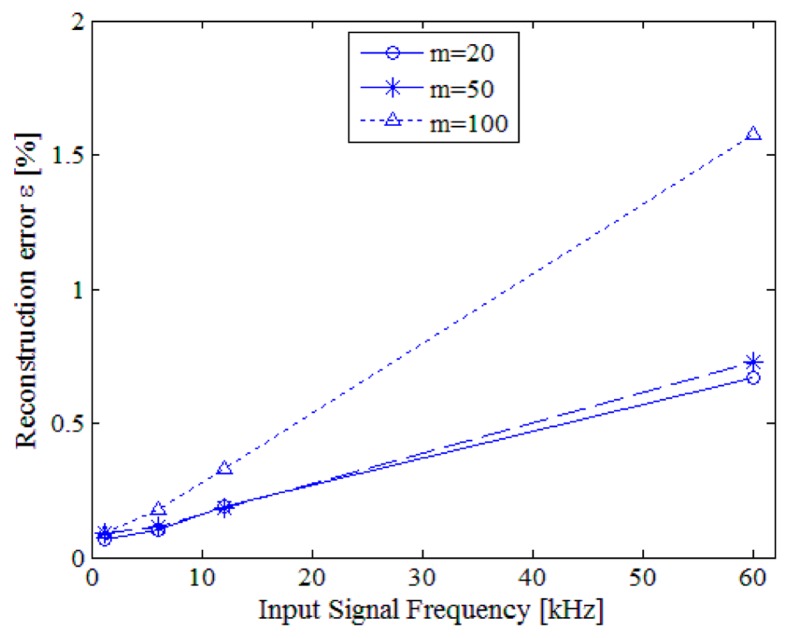
Reconstruction error *versus* the input signal frequency for different number *m* of randomly acquired samples.

More exhaustive tests have been carried out on pure sinusoidal signals in different conditions of input signal frequency *f_s_*, number of acquired samples *m*, ADC actual sample rate *f_conv_* and frequency ratio *f_Ck_/f_c_*. For each test configuration, 100 acquisitions have been made and the reconstruction error has been evaluated in terms of its mean and standard deviation values. In order to compare the results of the different configurations, the same sequence of random sampling instants has always been adopted. As an example, Figure 14 shows some results obtained when *T_conv_* and *f_c_* were equal respectively to 2.7 μs and 12 MS/s. Similar results have been gained in the other tests configurations. The reconstruction error worsened for higher values of input signal frequency; the main reason for this was the effect of the instruction cycle jitter, as it can be noticed in Figure 14 and Table 5.

**Figure 15.**
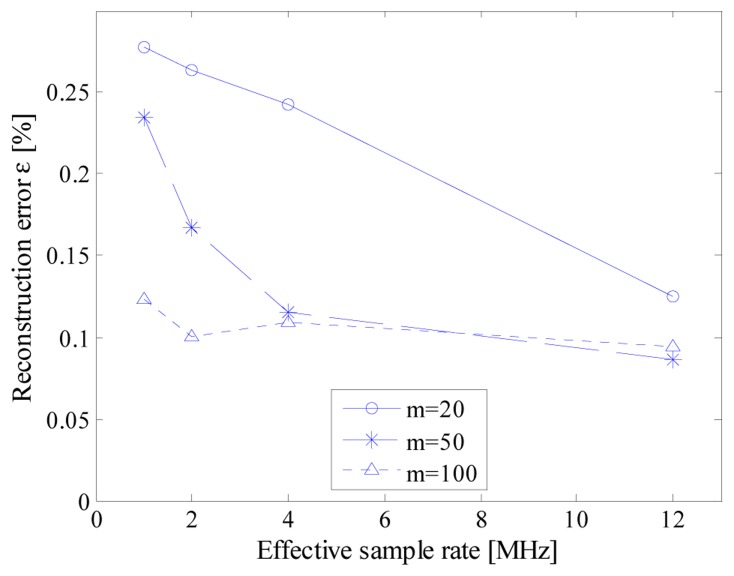
Reconstruction error *versus* the effective sample rate for different number *m* of randomly acquired samples.

In particular, Figure 15 reports the evolution of the mean reconstruction error *versus* the effective sample rate of the converter for 6 kHz input signal; as it can be noticed, the higher the effective sample rate, the better the reconstruction error, since the jitter effect is reduced. The jitter effect is more evident from the results reported in Table 5, which refer to a sinusoidal signal with frequency equal to 60 kHz, while the fundamental instruction clock adopted for the time-basis was equal either to 12 and 72 MS/s.

**Figure 16.**
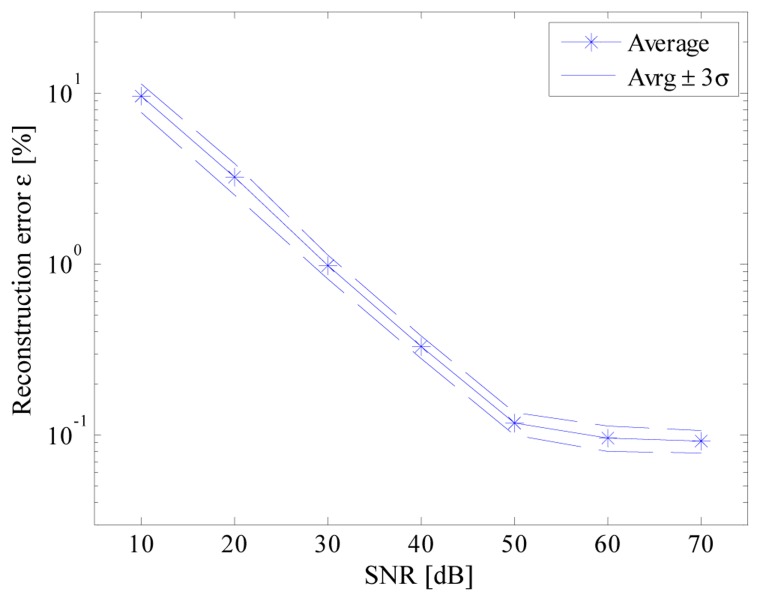
Reconstruction error *versus* signal-to-noise ratio.

A specific feature of the arbitrary function generator has been exploited to assess the performance of the acquisition strategy in the presence of noisy signals. To this aim, wideband AWGN signals (characterized by different amplitude levels and 240 MHz bandwidth) have been generated and added to the signal of interest. Figure 16 shows the results obtained when *T_conv_* and *f_c_* were equal, respectively, to 2.7μs and 12 MS/s; results similar to those achieved without noise are granted only for SNR higher than 50 dB, *i.e.*, the SNR value corresponding to the effective quantization noise of the adopted ADC as verified by the authors.

**Figure 17.**
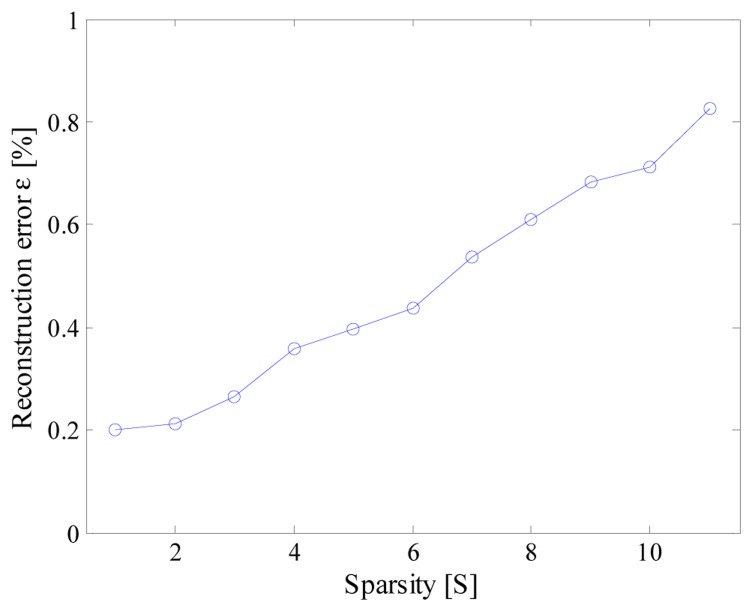
Reconstruction error *versus* the sparsity of the signal.

The effect of the input sparsity has, finally, been investigated by means of the aforementioned multisine signal. To this aim, signals composed by different harmonic components have been taken into account. As an example, the results obtained for input signal involving up to 11 spectral components when *T_conv_* and *f_c_* were equal respectively to 444 ns and 12 MS/s, which are given in Figure 17, show that the higher the spectral content, the worse the reconstruction error. Nevertheless, satisfying results (ε < 1%) are granted in the whole analysis range.

### 5.2. Tests Conducted on 8-Bits Microcontroller

Further experiments have been carried out on PIC18F4620 by Microchip, a typical example of very low cost, low performance 8-bit microcontroller. It is characterized by a maximum instruction cycle frequency *f_Ck_* equal to 10 MHz, data memory depth of 4 kB, a single ADC with selectable vertical resolution (8- and 10-bit) and full scale of 5 V [31].

With regard to the considered configuration, a traditional external 4 MHz clock has been adopted, thus granting an effective *f_Ck_* equal to 1 MHz. Similarly to the 32-bits microcontrollers, the nominal conversion interval *T_Conv_*is given by the sum of a constant term equal to 11 *T_Ck_* (needed for the digitization of the single sample) and a tunable sampling time ranging from 2 up to 20 *T_Ck_* [31]. Tests have been conducted to the minimum *T_conv_* (*i.e.*, 15 μs) capable of assuring reliable conversion of the input signal, thus granting a theoretical maximum sample rate of about 66 kS/s. Unfortunately, the actual sample rate was limited to 20 kS/s due to some instructions (beyond the traditional registers move) needed to implement the random acquisition strategy. Even in the presence of highly optimized assembly implementation of the code, no new acquisitions could start before the considered instructions have been executed. Thanks to the CS-based approach, the considered drawback has not only been recovered, but also overcome with sample rate values otherwise unavailable on the device.

A first set of measurements involved different conditions of input signals frequency and number of acquired samples. Experiments have been conducted at an effective sampling rate *f_c_* equal to 1 MS/s (*i.e.*, the worst condition in terms of instruction jitter) and nominal vertical resolution *n_bit_* equal either to 8- or 10-bits. As for the successive tests, signal amplitude has been set to match the ADC full scale (5V). For each test configuration, 100 successive random sequences have been acquired; as for the 32-bits microcontroller, the same sequences of sampling instants have been adopted in order to compare the performance throughout the different configurations. Some results, in terms of average reconstruction error and experimental standard deviation, are reported in Tables 6 and 7 for 10- and 8-bits resolution, respectively.

As it could be expected, the highest the number of acquired samples, the better the reconstruction performance. This is particularly true for signals characterized by the highest frequency values, in correspondence of which the effect of instruction cycle jitter proved to be worse; jitter influence was so high in such conditions that similar results have been achieved for both vertical resolutions. As for the 32-bits microcontroller, its effect should be mitigated by setting higher values of instruction cycle frequencies.

The effect of noise on reconstruction performance has then been assessed by means of several tests conducted with different levels of AWGN signals and number of acquired samples. The test signal was a pure sinusoidal tone whose frequency has been set to 100 Hz. As an example, some results obtained for SNR values varying upon the interval from 20 up to 50 dB are shown in Table 8. As it can be noticed, obtained results are better than those achieved in tests conducted through numerical simulations. Experienced improvement mainly relies on the reduced input bandwidth of the considered ADC (few tens of kHz), capable of cutting most of the high bandwidth (240 MHz) added noise.

**Figure 18.**
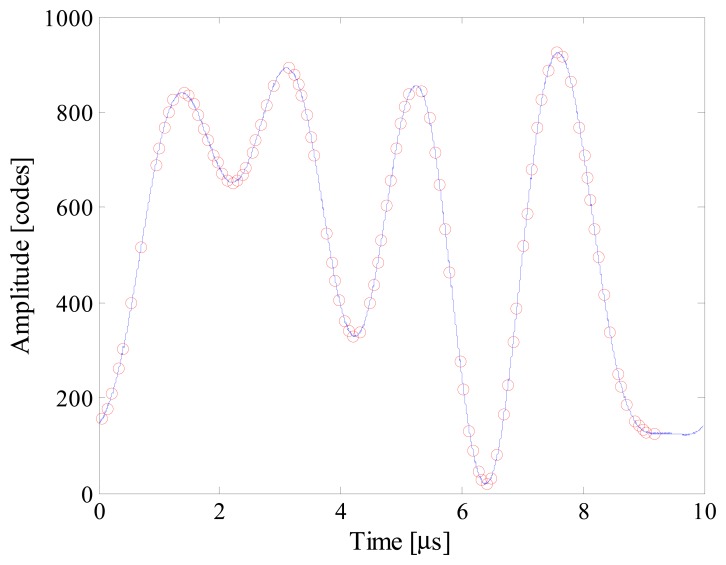
Example of estimated input signal, acquired samples (red circles), and reconstructed signal in the presence of 5-components multisine.

**Figure 19.**
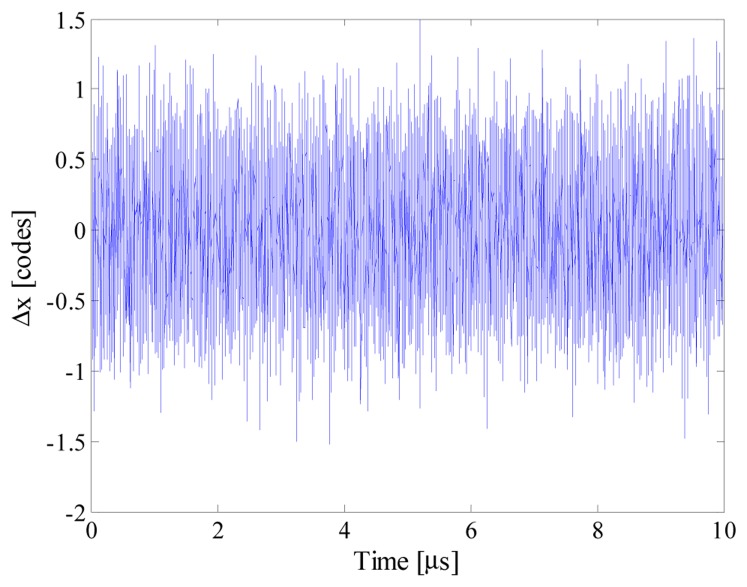
Point-by-point differences between reconstructed and input signal.

Further tests have finally been conducted with optimized *multisine* input signals. Different conditions of signal sparsity and number of acquired samples have been taken into account. As an example, Figure 18 shows estimated input signal, acquired samples and the reconstructed signal when *S* and *m* were equal respectively to 5 and 100. More details can be appreciated in Figure 19, where the point-by-point differences between reconstructed and input signal is plotted. Some results are given in Table 9. No reliable results were obtained with a number of acquired samples lower than 20, while 50 samples allowed to reconstruct the input signal with no more than 7 spectral components; as it can be expected, the best results were achieved only when at least 100 random samples were acquired.

## 6. Conclusions

The paper presented a new acquisition strategy, based on compressive sampling, for the improvement of the effective sampling rate of ADC usually integrated in microcontrollers or embedded systems. The proposed strategy exploits the availability of a high-resolution time-basis to finely set the sampling instants of the random samples acquired through a low-rate ADC. Thanks to the adopted CS approach, the signal of interest can be reconstructed with the same time-basis, thus enhancing the sample rate.

Preliminary tests conducted in simulations highlighted the promising effective performance of the proposed strategy in the presence of signals characterized by different amplitude and spectral contents. Experimental tests have also been carried out on microcontrollers characterized by different internal architecture and operating specifications. It is worth highlighting that satisfying results were obtained with both embedded systems, with increase of effective sample rate up to 50 times with respect to the actual ADC sample rate. Different measurement conditions in terms of input signal and noise have been taken into account as well as several configurations of acquisition parameters, such as effective sample rate, nominal number of bits and number of acquired random samples. The results obtained and discussed can be adopted as guidelines in choosing the proper trade-off between desired reconstruction error and computational burden.

Ongoing activities are mainly related to (i) the performance comparison, in terms of computational burden, among the different tools available for the solution of the optimization problem (10); (ii) the identification of optimal random sequence capable of making the proposed strategy with the lowest reconstruction error and (iii) the application of the proposed acquisition strategy on ADC characterized by input bandwidth greater than maximum sample rate [16].

## Figures and Tables

**Table 1.** Parameters values typically adopted in numerical tests.

**Parameter**	**Value**
number of Acquired Samples	80
number or reconstructed samples	10,000
*f_conv_* [kS/s]	10
*n_bit_*	12
*f_c_* [MS/s]	1
input signal frequency [kHz]	5
input signal amplitude [Codes]	2*^n_bit_^* − 1

**Table 2.** Parameters adopted in numerical tests conducted with different values of signal sparsity *S* and *m*.

**Parameter**	**Value**
number of acquired samples *m*	[20, 40, 60, 80, 100]
jitter [instruction cycles]	2
SNR [dB]	50
*f_Ck_*/*f_c_*	20
input signal sparsity *S*	[1,3,5,7,9,11]

**Table 3.** Reconstruction error, expressed in relative percentage value, obtained in numerical tests conducted with different values of signal sparsity *S* and *m*.

	***m* = 20**	***m* = 40**	***m* = 60**	***m* = 80**	***m* = 100**	
*S* = 1	0.02	0.01	0.01	0.01	0.01	Minimum
	1.04	0.85	0.80	0.78	0.79	Average
	3.52	1.89	1.65	1.55	1.61	Maximum

*S* = 3	0.28	0.05	0.05	0.05	0.05	Minimum
	10.86	1.32	0.59	0.54	0.52	Average
	22.45	11.88	1.56	1.38	1.32	Maximum

*S* = 5	4.11	0.21	0.04	0.05	0.03	Minimum
	17.34	6.77	1.00	0.45	0.41	Average
	35.52	20.45	10.06	1.71	1.29	Maximum

S = 7	12.15	1.41	0.25	0.11	0.08	Minimum
	21.55	13.28	5.69	0.61	0.38	Average
	51.58	22.71	17.26	2.47	1.11	Maximum

*S* = 9	11.59	9.05	1.19	0.19	0.12	Minimum
	26.98	17.09	10.82	3.40	0.45	Average
	50.76	28.47	24.22	16.41	1.94	Maximum

*S* = 11	11.61	9.06	5.82	0.90	0.19	Minimum
	28.10	19.15	14.49	8.34	2.28	Average
	56.48	26.18	25.37	19.26	9.48	Maximum

**Table 4.** Effect of *T_Conv_* (expressed in terms of multiple of fundamental instruction clock *t_Ck_*), on mean reconstruction errors and experimental standard deviation for different conditions of effective sample rate.

	**m =20**			**m =50**			**m = 100**			**t_Conv_ [t_Ck_]**
		
	**14**	**32**	**194**	**14**	**32**	**194**	**14**	**32**	**194**	
f_c_ = 1 MS/s	0.278	0.057	0.076	0.235	0.078	0.079	0.124	0.083	0.080	Mean
	0.068	0.008	0.019	0.047	0.024	0.021	0.021	0.008	0.007	Standard deviation

f_c_ = 2 MS/s	0.267	0.075	0.066	0.168	0.079	0.081	0.100	0.081	0.079	Mean
	0.053	0.017	0.009	0.021	0.015	0.012	0.014	0.015	0.009	Standard deviation

f_c_ = 4 MS/s	0.243	0.073	0.060	0.116	0.075	0.072	0.109	0.081	0.078	Mean
	0.045	0.019	0.010	0.057	0.006	0.010	0.059	0.006	0.003	Standard deviation

f_c_ = 12 MS/s	0.125	0.069	0.069	0.087	0.081	0.089	0.094	0.079	0.083	Mean
	0.051	0.016	0.012	0.047	0.011	0.009	0.021	0.011	0.007	Standard deviation

**Table 5.** Mean reconstruction errors and experimental standard deviation for different conditions of effective sample rate and fundamental instruction cycle clock.

	**m = 20**	**m = 50**	**m = 100**	
f_c_ = 1 MS/s f_Ck_ = 12 MHz	6.5	1.94	1.85	Mean
	1.2	0.34	0.30	Standard deviation
f_c_ = 1 MS/s f_Ck_ = 72 MHz	3.3	0.97	0.93	Mean
	0.9	0.12	0.13	Standard deviation
f_c_ = 2 MS/s f_Ck_ = 12 MHz	4.4	1.18	1.12	Mean
	0.8	0.12	0.11	Standard deviation
f_c_ = 2 MS/s f_Ck_ = 72 MHz	2.5	0.76	0.72	Mean
	0.6	0.08	0.07	Standard deviation
f_c_ = 4 MS/s f_Ck_ = 12 MHz	3.2	1.13	1.08	Mean
	0.6	0.11	0.10	Standard deviation
f_c_ = 4 MS/s f_Ck_ = 72 MHz	2.1	0.72	0.68	Mean
	0.4	0.13	0.10	Standard deviation
f_c_ = 12MS/s f_Ck_ = 12 MHz	1.9	0.71	0.73	Mean
	0.4	0.08	0.07	Standard deviation
f_c_ = 12 MS/s f_Ck_ = 72 MHz	1.6	0.62	0.67	Mean
	0.3	0.06	0.05	Standard deviation

**Table 6.** Mean reconstruction errors and experimental standard deviation with nominal vertical resolution of 10-bits.

**Signal Frequency (Hz)**	**m = 20**	**m = 50**	**m = 100**	
100	0.47	0.32	0.04	Mean
	0.16	0.10	0.01	Standard deviation

500	2.9	2.3	0.28	Mean
	0.2	0.2	0.01	Standard deviation

1000	4.4	4.4	0.55	Mean
	0.4	0.3	0.02	Standard deviation

**Table 7.** Mean reconstruction errors and experimental standard deviation with nominal vertical resolution of 8-bits.

**Signal Frequency (Hz)**	**m = 20**	**m = 50**	**m = 100**	
100	0.48	0.36	0.18	Mean
	0.15	0.09	0.01	Standard deviation

500	2.9	2.2	0.32	Mean
	0.3	0.2	0.03	Standard deviation

1000	4.4	4.3	0.58	Mean
	0.4	0.4	0.03	Standard deviation

**Table 8.** Mean reconstruction errors and experimental standard deviation in different noise conditions.

**SNR (dB)**	**m = 20**	**m = 50**	**m = 100**	
20	1.5	1.5	1.5	Mean
	0.2	0.2	0.2	Standard deviation
30	0.62	0.58	0.52	Mean
	0.10	0.11	0.05	Standard deviation
40	0.51	0.36	0.20	Mean
	0.15	0.10	0.03	Standard deviation
50	0.53	0.35	0.14	Mean
	0.14	0.09	0.02	Standard deviation

**Table 9.** Mean reconstruction errors and experimental standard deviation in different conditions of input signal sparsity.

**Sparsity**	**m = 50**	**m = 100**	
3	0.60	0.09	Mean
	0.22	0.01	Standard deviation

5	2.5	0.29	Mean
	0.8	0.04	Standard deviation

7	4.6	0.35	Mean
	1.2	0.11	Standard deviation

9	-	0.53	Mean
	-	0.16	Standard deviation

11	-	0.62	Mean
	-	0.21	Standard deviation
